# Patterns and predictors of cultural competence practice among Nigerian hospital-based healthcare professionals

**DOI:** 10.1186/s12909-023-04910-0

**Published:** 2023-12-08

**Authors:** Michael O. Ogunlana, Olufemi O. Oyewole, Joseph A. Aderonmu, Ogochukwu Kelechi Onyeso, Ayobamigbe Y. Faloye, Pragashnie Govender

**Affiliations:** 1 Department of Physiotherapy, Federal Medical Centre Abeokuta, Abeokuta, Ogun State Nigeria; 2 College of Health Sciences, University of KwaZulu-Natal, Private Bag X54001, Durban, South Africa; 3 Department of Physiotherapy, Olabisi Onabanjo University Teaching Hospital, Sagamu, Nigeria; 4 Department of Medical Rehabilitation, Obafemi Awolowo University, Ile-Ife, Nigeria; 5 Population Studies in Health, Faculty of Health Sciences, University of Lethbridge, Lethbridge, AB Canada; 6 Unit of Planning Research and Statistics, Federal Medical Centre Abeokuta, Abeokuta, Ogun State Nigeria

**Keywords:** Cultural competence, Healthcare professionals, Knowledge, Multicultural, Practice

## Abstract

**Background:**

Being culturally competent would enhance the quality of care in multicultural healthcare settings like Nigeria, with over 200 million people, 500 languages, and 250 ethnic groups. This study investigated the levels of training and practice of cultural competence among clinical healthcare professionals in two purposively selected Nigerian tertiary hospitals.

**Methods:**

The research was a cross-sectional study. A multi-stage sampling technique was used to recruit participants who completed the adapted version of Cultural Competence Assessment Instrument (CCAI-UIC). Data were analysed using descriptive statistics, Pearson’s correlation, ANOVA, and multivariate linear regression.

**Results:**

The participants were mainly women (66.4%), aged 34.98 ± 10.18 years, with ≤ 5 years of practice (64.6%). Personal competence had a positive weak correlation with age (*p* < 0.001), practice years (*p* = 0.01), training (*p* = 0.001), practice (*p* < 0.001), and organisational competence (*p* < 0.001). There were significant professional differences in the level of training (*p* = 0.005), and differences in training (*p* = 0.005), and personal competence (*p* = 0.015) across levels of educational qualifications. Increasing practise years (*p* = 0.05), medical/dental profession relative to nursing (*p* = 0.029), higher personal (*p* = 0.013), and organisational (*p* < 0.001) cultural competences were significant predictors of the level of training. Male gender (*p* = 0.005), higher years in practice (*p* = 0.05), local language ability (*p* = 0.037), rehabilitation professionals relative to nursing (*p* = 0.05), high culturally competent practice (*p* < 0.001), higher training opportunities (*p* = 0.013), and higher organisational competence (*p* = 0.001) were significant predictors of higher personal competence.

**Conclusion:**

About a third of the participants had no formal training in cultural competence. Incorporating cultural competence in the Nigerian healthcare professionals’ education curricula may enhance the quality of care in the multicultural setting.

**Supplementary Information:**

The online version contains supplementary material available at 10.1186/s12909-023-04910-0.

## Background

Cultural competence in healthcare professionals (HCPs) refers to their ability to understand and effectively communicate with individuals from diverse cultural backgrounds [1]. This skill is critical in Africa, where healthcare providers encounter patients from various ethnicities, languages, and belief systems. The growth of multicultural societies, characterised by diverse ethnic communities and socio-cultural systems, has necessitated cross-cultural contacts between HCPs and patients [1]. As such, cultural competence, which considers the role of culture, language, diversity, and racial and ethnic differences, has become essential in clinical settings [2, 3]. Cultural competence encompasses knowledge, attitudes and skills that enable cross-cultural communication and efficient interaction [4]. In healthcare, cultural competence is exhibited when practitioners understand and respect differences in health beliefs and behaviours, acknowledge and accredit variations within cultural groups, and adapt their practice to provide effective interventions for people from different ethnic backgrounds [5]. In newer contexts, cultural competence includes skills that enable a healthcare professional to navigate socio-cultural factors [6], including recognising and reconciling communication styles of patients from diverse cultural backgrounds and their significance in managing their illnesses or disease conditions [7, 8].

The global increase in international migration, cultural diversity, race and identities has necessitated clinical cultural competence in several countries to improve healthcare accessibility, satisfaction [9, 10], and quality [11]. Studies have shown that unconscious biases against other cultures or races can negatively impact patient care and lead to poor patient ratings of care [12, 13]. Consequently, scholars have emphasised integrating cultural competence in healthcare training to enhance knowledge, skills, awareness and attitudes that will foster quality healthcare delivery, especially among culturally diverse populations [14, 15]. Medical education programmes in the United States are incorporating cultural competence in healthcare [16]. Similarly, regions with amalgamation of unique cultures and ethnic and racial groups, such as in Europe, Asia [17–19], and Africa [20–23], are making efforts towards increasing multicultural competence in clinical settings.

Several studies have highlighted the significance of cultural competence in HCPs in Africa. For example, a study conducted by de Beer and Chipps [24] in South Africa found that healthcare professionals with non-English-speaking backgrounds have higher levels of cultural competence than English-speaking professionals and were more likely to provide patient-centred care and have better communication with patients from different cultural backgrounds. In addition, a study conducted by Matthews and Van Wyk [23] in South Africa revealed that the ability to speak more than one language facilitates acquiring cultural competence training for HCPs. This may positively influence their attitudes, knowledge, and skills in providing care to culturally diverse patients. The training may help them better understand patients’ cultural beliefs, norms, and practices, improving patient-provider relationships and health outcomes. Another study by Aragaw and colleagues [20] in Ethiopia emphasised the importance of cultural competence in maternal healthcare. The researchers found that HCPs who received in-service culturally competent training related to maternal health were four times more competent than those who were not. They were successful in establishing rapport, engaging patients in treatment, and providing service satisfaction in diverse communities.

In Nigeria, a country with over 250 ethnic groups and more than 500 distinct languages [25], the cultural competence of HCPs is critical. Although English is the official language, it is spoken at home by a small portion (7.2%) of the population [26]. Socio-cultural factors still persist and influence health-seeking behaviours by the citizens and the discharge of health services by the healthcare systems [27–30]. Additionally language and communication barriers remain pivotal in influencing the satisfaction and patient-reported outcomes in healthcare provider and patient interactions [31, 32]. Moreover, there are diverse health belief systems and ideologies among Nigerian cultural groups [33, 34], which may interfere with conventional medical practice, primarily when such medical services are not channelled through sound cultural competence. These pieces of evidence suggest the need to assess the HCPs training and practice of cultural competence in delivering health services in Nigeria.

Despite the clear need for the practice of culturally competent healthcare across global populations [8, 11, 35], there is a dearth of studies that precisely assess the training and practice of cultural competence among HCPs in Nigeria. Also, the culturally diverse setting of Nigeria [36] and the continuous rural-urban migration that occurs in the country [37] exposes HCPs to regular clinical encounters with patients from cultural, language, and ethnic orientations different from theirs. Moreover, a recent study found that Nigerians mistrust the Nigerian health system due to a lack of patient-centred care [38]. This finding from this study indeed suggests the need for cultural competence among health care providers in the Nigerian health system since patient-centred care and cultural competence go hand-in-hand. Thus, the present study investigated the levels of training and practice of cultural competence among clinical HCPs in Nigeria. The research questions are:

i. What are the levels of training, practice, personal and organisational cultural competence reported by the participants?
ii. What is the level of correlation between participants’ age, years of practice, levels of training, practice, personal and organisational cultural competencies?
iii. What are the differences in levels of formal training, personal competence, and practice across health professions and educational qualifications?
iv. What are the sociodemographic and professional predictors of the levels of formal training, personal competence, and practice of cultural competence among the participants?

## Methods

### Study design, participants and setting

An analytic cross-sectional study design was employed to assess the cultural competence of Nigerian health professionals. The study protocol was approved by the Federal Medical Centre Abeokuta and Olabisi Onabanjo University Teaching Hospital Health Research Ethics Committees, Nigeria (reference number: FMCA/470/HREC/01/2022/08; OOUTH/HREC/517/2022AP). The study was conducted in adherence to the ethical principles of the Declaration of Helsinki [39] and reported in line with Strengthening the Reporting of Observational Studies in Epidemiology (STROBE) guidelines [40].

The participants (HCPs) were selected who directly interact with patients. These were medical/dental practitioners, nurses, pharmacists, physiotherapists, and occupational therapists. To be included, the participant signed an informed consent, held an entry-level qualification, completed postgraduation internship where necessary, had current practice licence, and practicing in any of the two designated tertiary hospitals. However, healthcare professionals who do not give direct care to patients were excluded from this study. The selected tertiary hospitals were Olabisi Onabanjo University Teaching Hospital (OOUTH), Sagamu and Federal Medical Centre (FMCA), Abeokuta, both in Ogun State, Nigeria.

### Sample size determination

The sample size was determined from each Centre using the formula [Z^2^ p (1 – p)/e^2^]/ {1 + (Z^2^ p (1 – p)/e^2^ N)}. Proportion (p) = 0.5, Error (Margin) [e] = 0.04, 95% Confidence Level Z-score (Z) = 1.959964 and eligible population size (clinicians who have contact with patients) [N] = 660. Therefore, the minimum sample size was 406.

### Sampling technique and procedure

After obtaining ethical approval, a multi-stage sampling technique was used to concurrently recruit healthcare professionals from both study sites. The different stages involved purposive selection of the study centres, proportionate sampling of selected health care professionals and consecutive sampling (as the HCPs became available to participate in the research) within the professional group of participants. Eligible participants were approached individually by the investigators and research assistance for recruitment into the study. The study instrument was distributed in-person.

First, the purpose of the study was explained to each participant, who then signed an informed consent form. Second, the sociodemographic and professional characteristics of the participants were collected using a biodata form. Finally, the modified Cultural Competence Assessment Instrument (CCAI-UIC) was self-administered, while the researchers stayed back to give any required clarification and collect the questionnaire after completion. This approach minimises incomplete data and loss of questionnaires.

### Instrument

The tool for this research was the adapted version of the Cultural Competence Assessment Instrument (CCAI-UIC) developed by Balcazar and colleagues [41]. The adaptation of the CCAI-UIC is to suit the ethnicity of Nigerian HCPs. Hence, the only alteration to the original questionnaire was to change the races to the major ethnic groups in Nigeria. The CCAI-UIC is a 24-item self-administered questionnaire that assesses cultural competence on a three-factor model of cognitive (awareness/knowledge, behavioural (skills), and contextual (organisational/support) components on a 6-point *Likert scale* (strongly disagree = 1 to agree strongly = 6). The three-factor components of the instrument were reported to demonstrate relatively strong reliability and equal proportions of variance with *Cronbach alpha (α)* ranges between 0.76 and 0.83 [42]. The current study CCAI tool yielded *Cronbach alpha (α)* of 0.78.

### Data analysis

The data analysis was completed using SPSS version 24 software (Chicago, IL, USA). The participants’ demographic characteristics and training in cultural competence in clinical practice were summarised using *descriptive statistics* – *frequency* (*percentage*). Aside from the demographic variables, all the items in the questionnaire (9–36) were positively (re)coded and analysed for domain membership through exploratory factor analysis using maximum likelihood extraction and varimax orthogonal rotation. Four distinct constructs emerged (a group of correlated items) with eigenvalue ≥ 1. Level of training = summation of participants’ response (yes = 1, no = 0) to items 9i to 9viii and 9ix_R. Practice = summation of means of items 11 and 12 (i to vii) on a 6-point Likert scale where items entered as “not applicable” were adjusted for using case-wise deletion. Personal competence = summation of items 13, 14R, 15, 17R, 20, 22, 24R, 26, 28, 29R, 30, 31, 33, 34, 35, and 36R on a 6-point Likert scale. Organisational competence = summation of the responses to questions 16R, 17, 18, 19, 21, 23R, 25R, 27R, and 32R on a 6-point Likert scale. Items with “R” were initially negatively worded in the questionnaire and have been reverse-coded for analysis. Participants’ training, practice, personal, and organisational competence level was estimated by dichotomising each scale such that scores less than 70% of the upper limit of the scale were regarded as poor, while ≥ 70% was good.

*Pearson’s product-moment correlation* was used to test the correlation among the age of participants, number of practice years, cultural competence domains of training, practice, and personal and organisational competencies (constructs). *One-way analysis of variance (ANOVA)* was used to determine any significant mean difference in the constructs across education levels and professional designations. Finally, *multiple linear regression* (simultaneous entry) was employed to identify significant predictors of each construct using sociodemographic and practice characteristics as predictor variables. The derived continuous variables were diagnosed and fixed for *Pearson’s correlation, ANOVA*, and *multiple linear regression* assumptions. Continuous variables were normally distributed, with less than 3.5% missing variables, there were no *univariate* and *multivariate outliers*, and assumptions of *linearity* and *multicollinearity* were met. However, the data violated Levene’s test for homogeneity of variances; therefore, the Games-Howell test was used for *post-ANOVA* pair-wise comparison. The alpha level was set at ≤ 0.05 for all the analyses.

## Results

### Demographic characteristics of participants

Five hundred questionnaires were administered across the two centres (FMCA- 46.4%, OOUTH- 53.6%) with a non-response of 13%. Table 1 shows the distribution of participants across study locations, gender, duration of practice, and educational qualification. Most participants (66.4%) were female HCPs between the age group 19–39 years (52.0%). Yorubas ethnic group (88.7%) were the majority, 64.6% of the participants had 0–5 years of job experience, 86% revealed that they could speak a local language besides English to patients. Over half (68%) of the participants had entry-level (Bachelor/HND) education. Nurses (43%) and medical/dental practitioners (37.2%) were the majority of the participants.

**Table 1:** Participants’ demographic characteristics (*n* = 435, *missing variable *n* ≠ 435)

Variable	Frequency (%)	Mean (SD)	Range
**Gender**			
Female	289 (66.4)		
Male	146 (33.6)		
Age group (years)*		34.98 (10.18)	19–60
19–34	226 (52.0)		
35–60	178 (40.9)		
**Ethnicity**			
Igbo	33 (7.6)		
Hausa	4 (0.9)		
Yoruba	386 (88.7)		
Others	12 (2.8)		
Practice years*		6.52 (7.84)	0–35
0–5	281 (64.6)		
6–10	61 (14.0)		
11–39	71 (16.3)		
**Can speak at least one local language**			
Yes	374 (86.0)		
No	61 (14.0)		
**Level of education**			
Bachelor/HND	296 (68.0)		
Masters	33 (7.6)		
Professional degrees /fellowships	101 (23.2)		
Doctorates	5 (1.2)		
**Profession**			
Medical/Dental	162 (37.2)		
Nursing	187 (43.0)		
Pharmacy	17 (3.9)		
Physiotherapy	62 (14.3)		
Occupational therapy	7 (1.6)		
**Formal training**		2.15 (1.79)	0–9
Poor	421 (96.8)		
Good	14 (3.2)		
Practice*		8.47 (1.92)	0–12
Poor	172 (39.5)		
Good	249 (57.2)		
**Personal competence**		67.95 (10.53)	16–96
Poor	204 (46.9)		
Good	231 (53.1)		
**Organisational competence**		30.55 (6.41)	9–54
Poor	376 (86.4)		
Good	59 (13.6)		

The source of training about cultural competence by the HCPs includes learning about cultural competence alongside other classes in 29.7%, and lessons about cultural competence during fieldwork in 30.1% while 32.6% knew this construct through interactions with other professionals on the job. About a third of the participants (33.3%) had no formal training in cultural competence (Table 2). Though many of the participants (66.6%) agreed that their organisation does not provide ongoing training on cultural competence, they agreed that they can learn from their ethnic minority clients (85.5%) (Table 3).

**Table 2:** Cultural competence training across professions (*n* = 435)

Variable		Frequency (%)^b^					
		Medical/ Dental 162 (100)	Nursing 187 (100)	Pharmacy 17 (100)	Physio- therapy 62 (100)	Occupational therapy 7 (100)	Total 435 (100)
I took a required class that focused specifically on this topic in school.	Yes	9 (5.6)	64 (34.2)	3 (17.6)	6 (9.7)	3 (42.9)	85 (19.5)
I took an elective class that focused specifically on this topic in school.	Yes	11 (6.8)	37 (19.8)	1 (5.9)	8 (12.9)	3 (42.9)	60 (13.8)
This topic was covered in various classes in school.	Yes	36 (22.2)	72 (38.5)	3 (17.6)	16 (25.8)	2 (28.6)	129 (29.7)
I learned about this during my fieldwork experience in school.	Yes	33 (20.4)	68 (36.4)	7 (41.2)	20 (32.3)	3 (42.9)	131 (30.1)
I took continuing education workshops or courses on this topic.	Yes	5 (3.1)	37 (19.8)	1 (5.9)	4 (6.5)	1 (14.3)	48 (11.0)
I gained knowledge from reading about this topic on my own.	Yes	25 (15.4)	47 (25.1)	7 (41.2)	23 (37.1)	4 (57.1)	106 (24.4)
I learned about it through supervision on the job.	Yes	34 (21.0)	36 (19.3)	3 (17.6)	12 (19.4)	5 (71.4)	90 (20.7)
I learned about it through inter-professional interaction at my workplace.	Yes	44 (27.2)	60 (32.1)	6 (35.3)	27 (43.5)	5 (71.4)	142 (32.6)
I have had no formal training in cultural competence.^a^	Yes	89 (54.9)	28 (15.0)	3 (17.6)	25 (40.3)	0 (0.0)	145 (33.3)

^a^Reverse-coded during inferential analysis. ^b^NO responses are [435 – frequency (100 - %)]

**Table 3:** Levels of the personal and organisational factors of cultural competence (n = 435)

Item	Missing	Strongly disagreed ------------> Strongly agreed						Mean (Median)
	-	1	2	3	4	5	6	
	*f* (%)	*f* (%)	*f* (%)	*f* (%)	*f* (%)	*f* (%)	*f* (%)	
**Personal factors**								
I feel that I can learn from my ethnic minority clients.	10 (2.3)	11 (2.5)	8 (1.8)	34 (7.8)	65 (14.9)	104 (23.9)	203 (46.7)	**5.0 (5)**
It is hard adjusting my therapeutic strategies with ethnic minority clients.^a^	7 (1.6)	82 (18.9)	89 (20.5)	62 (14.3)	80 (18.4)	74 (17.0)	41 (9.4)	**3.2 (3)**
I am effective in my verbal communication with clients whose culture is different from mine.	2 (0.5)	23 (5.3)	47 (10.8)	73 (16.8)	91 (20.9)	117 (26.9)	82 (18.9)	**4.1 (4)**
I do not consider the cultural backgrounds of my clients when food is involved.^a^	6 (1.4)	230 (52.9)	62 (14.3)	39 (9.0)	41 (9.4)	29 (6.7)	28 (6.4)	**2.2 (1)**
I feel confident that I can learn about my clients' cultural backgrounds.	6 (1.4)	14 (3.2)	21 (4.8)	31 (7.1)	72 (16.6)	123 (28.3)	168 (38.6)	**4.8 (5)**
I am effective in my nonverbal communicsation with clients whose culture is different from mine.	1 (0.2)	32 (7.4)	32 (7.4)	52 (12.0)	96 (22.1)	128 (29.4)	94 (21.6)	**4.2 (5)**
I feel that I have limited experience working with ethnic minority clients.^a^	3 (0.7)	85 (19.5)	64 (14.7)	88 (20.2)	77 (17.7)	83 (19.1)	35 (8.0)	**3.3 (3)**
I am sensitive to valuing and respecting differences between my cultural background and my clients' cultural heritage.	3 (0.7)	31 (7.1)	19 (4.4)	34 (7.8)	61 (14.0)	126 (29.0)	161 (37.0)	**4.7 (5)**
I have opportunities to learn culturally responsive behaviours from peers.	2 (0.5)	25 (5.7)	33 (7.6)	69 (15.9)	90 (20.7)	125 (28.7)	91 (20.9)	**4.2 (4)**
I do not feel that I have the skills to provide services to ethnic minority clients.^a^	2 (0.5)	150 (34.5)	100 (23.0)	44 (10.1)	71 (16.3)	43 (9.9)	25 (5.7)	**2.6 (2)**
I examine my own biases related to race and culture that may influence my behaviour as a service provider.	5 (1.1)	63 (14.5)	39 (9.0)	69 (15.9)	74 (17.0)	121 (27.8)	64 (14.7)	**3.8 (4)**
I actively strive for an atmosphere that promotes risk-taking and self-exploration.	7 (1.6)	44 (10.1)	26 (6.0)	67 (15.4)	95 (21.8)	133 (30.6)	63 (14.5)	**4.0 (4)**
I would find it easy to work competently with ethnic minority clients.	2 (0.5)	24 (5.5)	38 (8.7)	74 (17.0)	65 (14.9)	116 (26.7)	116 (26.7)	**4.3 (5)**
I openly discuss other issues I may have in developing multicultural awareness.	5 (1.1)	45 (10.3)	37 (8.5)	76 (17.5)	108 (24.8)	94 (21.6)	70 (16.1)	**3.9 (4)**
I learn about different ethnic cultures through educational methods or life experiences.	1 (0.2)	20 (4.6)	20 (4.6)	40 (9.2)	84 (19.3)	140 (32.2)	130 (29.9)	**4.6 (5)**
It is difficult for me to accept that religious beliefs may influence how ethnic minorities respond to illness and disability.^a^	1 (0.2)	157 (36.2)	87 (20.0)	48 (11.1)	52 (12.0)	42 (9.7)	48 (11.0)	**2.7 (2)**
**Organisational factors**								
My organisation does not provide ongoing training on cultural competence.^a^	4 (0.9)	65 (14.9)	23 (5.3)	53 (12.2)	40 (9.2)	65 (14.9)	185 (42.5)	**4.3 (5)**
I receive feedback from supervisors on how to improve my practice skills with clients from different ethnic minority backgrounds.	7 (1.6)	92 (21.1)	51 (11.7)	66 (15.2)	62 (14.3)	95 (21.8)	62 (14.3)	**3.5 (4)**
s At work, pictures, posters, printed materials, and toys reflect the culture and ethnic backgrounds of ethnic minority clients.	5 (1.1)	133 (30.6)	66 (15.2)	61 (14.0)	46 (10.6)	48 (11.0)	76 (17.5)	**3.1 (3)**
Cultural competence is included in my workplace's mission statement, policies, and procedures.	6 (1.4)	97 (22.3)	57 (13.1)	77 (17.7)	62 (14.3)	85 (19.5)	51 (11.7)	**3.3 (3)**
The way services are structured in my setting makes it difficult to identify the cultural values of my clients.^a^	5 (1.1)	105 (24.1)	71 (16.3)	81 (18.6)	67 (15.4)	75 (17.2)	31 (7.1)	**3.1 (3)**
It is difficult to practice skills related to cultural competence.^a^	6 (1.4)	89 (20.5)	66 (15.2)	99 (22.8)	87 (20.0)	52 (12.0)	36 (8.3)	**3.1 (3)**
My workplace does not support using resources to promote cultural competence.^a^	2 (0.5)	107 (24.6)	83 (19.1)	85 (19.5)	60 (13.8)	50 (11.5)	48 (11.0)	**3.0 (3)**
My workplace does not support my participation in the cultural celebrations of my clients.^a^	5 (1.1)	148 (34.0)	93 (21.4)	63 (14.5)	53 (12.2)	38 (8.7)	35 (8)	**2.6 (2)**

Participants’ responses on a continuum of 1 = Strongly disagree (SD) through 6 Strongly Agreed (SA) considering their work over the past year. ^a^Reverse-coded during inferential analysis

Table 4 shows *Pearson’s correlations* between participants’ age, practice years, levels of training, practice, and competence domains. Age of the participants was significantly correlated with years of practice (*p* = 0.001), level of cultural competence practice (*p* = 0.001) and personal competence (*p* = 0.001). Years of practice were significantly correlated with the level of cultural competence practice (*p* = 0.012) and personal competence (*p* = 0.01). Levels of training in cultural competence were significantly correlated with both personal (*p* = 0.001) and organisational (*p* = 0.001) competencies.

**Table 4:** Pearson’s correlations between participants’ age, practice years, levels of training, practice, and competence domains

Variable	Practice years	Level of training	Level of practice	Personal competence	Organisational competence
**Age of respondent**	*r* = 0.76	*r* = -0.03	*r* = 0.17	*r* = 0.19	*r* = -0.04
	*p* < 0.001^a^	*p* = 0.600	*p* = 0.001^a^	*p* < 0.001^a^	*p* = 0.471
**Practice years**		*r* = -0.02	*r* = 0.13	*r* = 0.13	*r* = 0.01
		*p* = 0.652	*p* = 0.012^a^	p = 0.010^a^	*p* = 0.770
**Level of training**			*r* = 0.08	*r* = 0.16	*r* = 0.24
			*p* = 0.125	*p* = 0.001^a^	*p* < 0.001^a^
**Level of practice**				*r* = 0.33	*r* = 0.03
				*p* < 0.001^a^	p = 0.606
**Personal competence**					*r* = 0.20
					*p* < 0.001^a^

^a^Pearson correlation (*r*) was significant at *p* < 0.05 level (2-tailed)

The *ANOVA* results in Table 5 show that there were significant differences in the training (*p* = 0.005) and personal competence (*p* = 0.015) across levels of education, but not practice (*p* = 0.164). Games-Howell post hoc test showed that participants with entry-level qualifications reported higher levels of training than those with fellowship (*mean difference* [*MD]* = 0.610, *p* = 0.003). There was no significant mean difference in training between other levels of education. However, those with postgraduate academic (master or doctoral) qualifications reported higher personal cultural competence than those with entry-level qualifications (*MD* = 0.940, *p* = 0.021). Similarly, there was a significant difference in cultural competence training across health professions (*p* = 0.005), such that nurses (*MD* = 0.636, *p* = 0.005) and physio/occupational therapists (*MD* = 0.655, *p* = 0.023) had higher training than medical/dental practitioners. There were no differences in the level of practice and personal competence across the health professions (Table 5).

**Table 5:** ANOVA: Differences in training, competence, and practice across educational levels and designations (*n* = 435)

Variable	Training		Personal competence		Practice	
	n	Mean ± SD	n	Mean ± SD	n	Mean ± SD
Educational level						
Entry-level (Bachelor & Diploma)	296	2.27 ± 1.77	296	8.32 ± 20	285	67.43 ± 10.86
Graduate academic (Master & Doctoral)	38	2.50 ± 2.25	38	9.26 ± 1.93	37	70.74 ± 9.79
Graduate professional (Fellowship)	101	1.66 ± 1.53	101	8.60 ± 1.61	99	68.45 ± 9.66
		*F*(2, 432) = 5.29 ***p*** **= 0.005** ^a^		*F*(2, 432) = 4.28 ***p*** **= 0.015** ^a^		*F*(2, 432) = 1.81 ***p*** **= 0.164**
**Profession**						
Medical/Dental	162	1.77 ± 1.51	157	8.67 ± 1.72	162	68.66 ± 10.15
Nurse	187	2.40 ± 2.02	178	8.39 ± 1.83	187	68.08 ± 10.69
Pharmacist	17	2.00 ± 1.58	17	8.10 ± 2.09	17	68.18 ± 8.77
Physio- & Occupational therapist	69	2.42 ± 1.60	69	8.31 ± 2.48	69	65.90 ± 11.27
		* F*(3, 431) = 4.41 ***p*** **= 0.005** ^a^		*F*(3, 417) = 1.07 ***p*** **= 0.364**		* F*(3, 431) = 1.33 ***p*** **= 0.335**

^a^ *F*-statistics were significant at *p* < 0.05 level (2-tailed)

*Multivariate linear regression* showing predictors of training opportunity, practice, and cultural competence among health professionals is shown in Table 6. Years of practice, medical/dental profession, personal competence, and organisational competence predicted the level of training. Personal competence was predicted by gender, years of practice, language of communication, rehabilitation professions, good practice, training opportunities, and organisational competence. Only medical/dental profession and personal competence predicted practice. With increased years of practice, health professionals are less likely to engage in cultural competence training (*p* = 0.050). Medical/dental professionals, in comparison with nurses, are less likely to have cultural competence training (*p* = 0.029). increase in personal (*p* = 0.013) and organizational (*p* < 0.001) competences encouraged more training in cultural competence. Men demonstrated higher personal competence compared to women (*p* = 0.005), and it increases with an increase in years of practice (*p* = 0.050) and the ability to communicate in both English and a local dialect (*p* = 0.037). Good practice (*p* < 0.001), increased training opportunity (*p* = 0.013) and better organisational competence (*p* = 0.001) increases personal competence. A rehabilitation professional is less likely to achieve personal competence than nurses (*p* = 0.050). Being a medical/dental professional (*p* = 0.036) improves the practice of cultural competence compared to nurses, while an increase in personal competence also improves practice (*p* < 0.001).

**Table 6:** Multivariate linear regression showing predictors of training opportunity, practice, and cultural competence among health professionals

Variable (reference)	Training		Personal competence		Practice	
	Standardised Coefficients (β)	*p*-value	Standardised Coefficients (β)	*p*-value	Standardised Coefficients (β)	*p*-value
Men (women)	-0.022	0.698	**0.152**	**0.005** ^a^	-0.061	0.274
Practice years (increase)	**-0.101**	**0.050** ^a^	**0.097**	**0.050** ^a^	0.098	0.065
Yoruba (non-local ethnicity)	0.016	0.751	-0.028	0.573	0.067	0.186
Federal hospital (State)	-0.052	0.330	0.030	0.561	0.027	0.615
English & a local dialect (English only)	-0.063	0.211	**0.100**	**0.037** ^a^	-0.029	0.560
Medical /Dental (Nurse)	**-0.160**	**0.029** ^a^	-0.037	0.602	**0.152**	**0.036** ^a^
Pharmacy (Nurse)	-0.058	0.252	0.020	0.681	-0.022	0.655
Physio- & Occupational therapist (Nurse)	0.024	0.682	**-0.111**	**0.050** ^a^	0.066	0.259
Graduate-MSc/Ph.D. (Bachelor)	0.050	0.342	0.028	0.580	0.060	0.245
Graduate Fellowship (Bachelor)	-0.026	0.677	0.017	0.781	-0.012	0.839
Good practice (increase)	0.030	0.553	0.291	**< 0.001** ^a^	-	-
Personal competence (increase)	**0.131**	**0.013** ^a^	-	-	**0.311**	**< 0.001** ^a^
Training opportunity (increase)	**-**	**-**	**0.120**	**0.013** ^a^	0.030	0.553
Organisational competence (increase)	**0.193**	**< 0.001** ^a^	**0.157**	**0.001** ^a^	-0.039	0.435

^a^Standardised regression coefficient (β) is significant at *p* ≤ 0.05

Model summary: Training, *F*(13, 387) = 3.88, *p *< 0.001, adjusted R^2^ = 0.086. Personal competence, *F*(13, 387) = 6.81, *p *< 0.001, adjusted R^2^ = 0.159. Practice, *F*(13, 387) = 4.54, *p *< 0.001, adjusted R^2^ = 0.103

## Discussion

The findings of this study revealed that a substantial number of hospital-based HCPs lacked formal training in cultural competence, suggesting the need for increased training and practice of cultural competence among healthcare professionals in Nigeria. Cultural competence is crucial in providing quality healthcare services to individuals from diverse cultural backgrounds, as it involves the understanding and respecting cultural differences, beliefs and practices. The fact that the results of this study reveal that about a third of the HCPs had no formal training in cultural competence indicates a gap in the healthcare education system in Nigeria. This may suggest a need for curriculum enhancements to include cultural competencies in student training. The differences in cultural competence training based on levels of education and the health profession being practised also raise considerations. It suggests that certain HCPs may require more emphasis on cultural competence training than others. In this case, nurses and physiotherapists/occupational therapists reported more training in cultural competence training than medical/dental practitioners. The identified predictors of level of training were more practise years, nursing relative to the medical/dental profession, and higher personal, and organisational cultural competencies. Overall, this study underscores the importance of prioritising cultural competence in practitioners for more culturally sensitive and appropriate care to individuals from diverse backgrounds, ultimately improving health care outcomes for all in Nigeria.

### Level of training in cultural competence

In this study, only 19.5% of participants took specific classes on cultural competence, while others relied on informal training and experience to become culturally competent healthcare providers. This finding aligns with a study involving HCPs specialised in diabetes care in Sweden, where 21% of the health professionals developed cultural competence through primary education [43]. Despite this, our results reveal some important facts: Firstly, many HCPs lack structured curriculum training in cultural competence, a situation not unique to Nigerian HCPs. In the United States, a study reported that a study reported 67% compliance with cultural competence training, and only two out of the 18 schools reviewed in the study had structured training curricula [44]. Secondly, participants in our study utilised various non-traditional training avenues (such as continuing education programmes, interaction with colleagues, fieldwork practice, and internships) to compensate for their lack of formal training in cultural competence. This demonstrates that formal training in cultural competence is not the sole requirement for providing culturally competent care. Our findings, along with previous studies [44, 45], emphasise the need for the deliberate inclusion of cultural competence in the curriculum of institutions training HCPs.

Moreover, our results provided evidence that participants with entry-level qualifications had significantly higher cultural competence training than those with graduate professional degrees (fellowships), suggesting that cultural competence is better as a foundational training requirement for HCPs [46, 47]. Higher levels of professional training may assume that communication, interaction, and cultural competence is simply a prerequisite that should have been fulfilled from lower levels of training. This is supported by the fact that nursing and physiotherapy/occupational therapy practitioners in our study had significantly higher cultural competence training than medical/dental professionals. As these professions have more intimate rapport and more extended interactions with patients, cultural competence may have been emphasised in their training or essential in achieving success with healthcare users [2, 48, 49]. However, overall, our results suggest an inadequate level of formal training in cultural competence across health professions in Nigeria, which requires urgent redress.

### Practise of cultural competence

The results of our study using the adapted CCAI-UIC revealed no significant difference in the practice of cultural competence among participants regardless of their educational levels or health professions. However, we found weak but significant correlations between the age and years of practice of respondents and their level of cultural competence practice. Based on these findings, we recommend enhancing formal training in cultural competence for all health professions [11, 50] regardless of the level of educational training, age and years of practice of practitioners.

Interestingly, our results also revealed that being a medical/dental professional is a significant predictor of the practice of cultural competence when compared to nurse professionals. Another study emphasised the importance of healthcare leadership in acquiring cultural competence, suggesting that formal leadership or playing a central role is crucial [51]. This finding indicates that job-specific requirements, especially healthcare leadership (which is usually assumed by medical/dental professionals in Nigeria) may demand higher practice of cultural competence from these professional. This is particularly relevant in Nigerian healthcare systems, where the role of the medical/dental professional hold significant importance within the healthcare team.

Similarly, we found that the practice of cultural competence increases with personal competence. Betancourt (54) concluded in his paper that the goal of cultural competence “is to assure that health care providers are prepared to provide quality care to diverse populations” [52]. Therefore, an increase in personal competence is expected to lead to better practice of cultural competence in healthcare settings.

### Personal and organisational levels of cultural competence

Our findings revealed that having a graduate academic degree (master’s and doctoral) significantly predicts the presence of a higher personal competence than having only an entry-level degree. Although graduate degrees acquiring more profound knowledge and competency in desired fields, growing emphasis on cultural competence over the years may have led to higher-level academic degree graduates gaining more personal competence. For example, a previous study reported that introducing cultural competence to a graduate program in occupational therapy curriculum sustained the cultural competence of the students engaged in the curriculum upon graduation [53]. It is plausible to suspect that HCPs who had graduate-level training must have had some form of exposure to cultural competency training, leading to higher personal competence. However, it must be stated that the result of our multiple linear regression did not provide evidence that a graduate degree is a significant predictor of higher personal competence compared to having only a bachelor’s degree. Thus, more studies are required to make more robust inferences from these results.

Furthermore, our results indicate a significant positive correlation between personal competence and organisational competence. We also found that increased organisational competence predicts personal competence. A study suggested that organisations’ support can help staff discharge culturally competent services [54]. Similarly, it has been reported that health institutions can improve professionals’ cultural competence by increasing the availability of information and access to cultural skills and materials relevant to the users of health services [43].

### Implications for policy and practice

Like many other African nations, Nigeria has diverse ethnicities or racial groups, and there are ongoing demographic changes in major cities in Africa. Cultural competency is essential in providing patient-centred care and optimal outcomes. Efforts should be directed at improving cultural competency among health professionals, since research has revealed inadequacies in cultural competence [20, 24]. This can be done or achieved through education at institutional, organisational, and individual levels. At the educational institution level, cultural competence should be part of clinical education rather than single seminars, practical skills should be taught, utilizing interactive educational methods such as role-play, standardized patient encounters, and self-reflective journal assignments [55]. Health care organizations can institutionalize cultural competence through identified three levels: the clinical level, structural level, and organisational level [56]. Organisational policy should encourage and mandate regular cultural competence training by providing time and resources to support the policy. At the individual health professional level, they should engage in culturally responsive learning for self-reflection, understanding, and uptake of the principles for changes in practice styles [56].

Improving cultural competency among health providers may foster good relationships between providers and patients and thereby enhance adherence to treatment recommendations leading to optimal health outcomes. Thus, organizational culture and organizational structures that promote and accept cultural competence training are warranted. The organization should encourage its staff to apply the cultural competence they have learnt. This may require economic incentive systems that reward culturally competent care [57].

### Limitations

This study has certain limitations that should be acknowledged. Firstly, the participants were exclusively from two tertiary health institutions in Nigeria. While these institutions cater to diverse cultural groups, it is important to note that a significant majority of participants belonged to the Yoruba ethnic group. This may introduce a potential bias in the findings. Additionally, the study did not consider the participant’s training institutions. Since HCPs in the country are trained from any of the 36 states and federal capital territory in the country and, in addition, overseas, it is possible that HCPs who received training either abroad or in certain institutions were exposed to more cultural competence training than others. To address these limitations, further studies should aim to include a more diverse range of healthcare institutions and consider the participants’ training institutions or countries. Furthermore, it is important to acknowledge the shift in focus from cultural competence to cultural humility and cultural safety. These concepts highlight the importance of healthcare providers actively seeking to understand and respect their clients’ cultural backgrounds. Therefore, we recommend that future studies explore the practice of cultural humility and safety in conjunction with cultural competence.

## Conclusion

In Nigeria, health professionals often work with patients from diverse cultural and ethnic backgrounds. However, many of these practitioners lack formal training in cultural competence. Although some individual health professionals and health organisations are making efforts towards addressing this issue, there is a clear necessity for a structured and mandatory curriculum on cultural competence in training for all Nigerian health professionals. Future work is recommended to assess barriers to practicing cultural competence among healthcare providers in the Nigerian health system.

### Supplementary Information

**Additional file 1.**

## Data Availability

The questionnaire used and datasets analysed during the current study are available from the corresponding author on reasonable request.

## Abbreviations

ANOVA: Analysis of variance
CCAI-UIC: Cultural Competence Assessment Instrument
FMCA: Federal Medical Centre, Abeokuta
HCPs: Health Care Professionals
HND: Higher National Diploma
MD: Mean difference
OOUTH: Olabisi Onabanjo University Teaching Hospital
