# Supplementary material for: Patterns and predictors of cultural competence practice among Nigerian hospital-based healthcare professionals
Source: BMC Med Educ. 2023 Dec 8;23:933. doi: 10.1186/s12909-023-04910-0 (PMC10709888; doi:10.1186/s12909-023-04910-0)
Supplement: Supplementary file 1 — Additional file 1. [file 12909_2023_4910_MOESM1_ESM.docx]

**APPENDIX I**

**CULTURAL COMPETENCE ASSESSMENT INSTRUMENT (CCAI)**

**PART 1: ABOUT YOU**

Please circle the numbers or complete the correct response(s) to the following questions.

1. Are you: (1) Female (2) Male

2. How old are you? ________ Years

3. What is your race/ethnicity? (Select ALL applicable):

(1) Igbo

(2) Hausa

(3) Yoruba

(4) Other. Please specify ______________________

4. Do you speak any language(s) other than English when providing services? (1) Yes (2) No

5. If Yes, what are these languages? __________________________________________

6. What is the highest level of education you have obtained?

(1) Bachelor's degree/HND

(2) Master's degree (MA, MS)

(3) Professional Degree/Fellowships

(4) Doctorate Degree (Ph.D./ Ed.D)

7. What is your profession?

(1) Medical or Dental Practitioner

(2) Nurse Practitioner

(3) Rehabilitation Practitioner (Please Specify) …….

8. How many years have you been working in your current position? ________ Years

9. Which of the following types of training did you receive on cultural competency, if any?

Circle ALL the responses that apply.

(1) I took a required class that focused SPECIFICALLY on this topic in school

(1) I took an elective class that focused SPECIFICALLY on this topic in school

(2) This topic was covered in various classes in school

(3) I learned about this during my fieldwork experience in school

(4) I took continuing education (CE) workshops or CE courses on this topic

(5) I gained knowledge from reading about this topic on my own

(6) I learned about it through supervision on the job

(7) I learned about it through interaction with professionals from other disciplines at my workplace

(8) I have had no formal training on cultural competence

**PART 2: ABOUT YOUR WORK during the PAST YEAR ONLY**

Please circle the number(s) or complete the correct response(s) to the following questions:

10. Indicate the top 3 populations that you see most often in your work:

(1) Igbo

(2) Yoruba

(3) Hausa

(4) Ijaw

(5) Kanuri

(6) Fulani

(7) Other. Please specify __________________________

11. Rate your level of success in outreaching (effectively working with) the following ethnic populations:

|  | Very successful |  |  |  |  | Very unsuccessful | N/A |
| --- | --- | --- | --- | --- | --- | --- | --- |
| Igbo | 6 | 5 | 4 | 3 | 2 | 1 | 0 |
| Hausa | 6 | 5 | 4 | 3 | 2 | 1 | 0 |
| Yoruba | 6 | 5 | 4 | 3 | 2 | 1 | 0 |
| Ijaw | 6 | 5 | 4 | 3 | 2 | 1 | 0 |
| Kanuri | 6 | 5 | 4 | 3 | 2 | 1 | 0 |
| Fulani | 6 | 5 | 4 | 3 | 2 | 1 | 0 |
| Other | 6 | 5 | 4 | 3 | 2 | 1 | 0 |

12. Rate your level of success in retaining the following ethnic populations:

|  | Very successful |  |  |  |  | Very unsuccessful | N/A |
| --- | --- | --- | --- | --- | --- | --- | --- |
| Igbo | 6 | 5 | 4 | 3 | 2 | 1 | 0 |
| Hausa | 6 | 5 | 4 | 3 | 2 | 1 | 0 |
| Yoruba | 6 | 5 | 4 | 3 | 2 | 1 | 0 |
| Ijaw | 6 | 5 | 4 | 3 | 2 | 1 | 0 |
| Kanuri | 6 | 5 | 4 | 3 | 2 | 1 | 0 |
| Fulani | 6 | 5 | 4 | 3 | 2 | 1 | 0 |
| Other | 6 | 5 | 4 | 3 | 2 | 1 | 0 |

|  | Considering Your Work Over the Past Year | Strongly agree |  |  |  |  | Strongly disagree |
| --- | --- | --- | --- | --- | --- | --- | --- |
| 13. | I feel that I can learn from my ethnic minority clients. | 6 | 5 | 4 | 3 | 2 | 1 |
| 14. | It is hard adjusting my therapeutic strategies with ethnic minority clients. | 6 | 5 | 4 | 3 | 2 | 1 |
| 15. | I am effective in my verbal communication with clients whose culture is different from mine | 6 | 5 | 4 | 3 | 2 | 1 |
| 16. | My organisation does not provide ongoing training on cultural competence. | 6 | 5 | 4 | 3 | 2 | 1 |
| 17. | I do not consider the cultural backgrounds of my clients when food is involved. | 6 | 5 | 4 | 3 | 2 | 1 |
| 18. | I receive feedback from supervisors on how to improve my practice skills with clients from different ethnic minority backgrounds. | 6 | 5 | 4 | 3 | 2 | 1 |
| 19. | At work, pictures, posters, printed materials and toys reflect the culture and ethnic backgrounds of ethnic minority clients. | 6 | 5 | 4 | 3 | 2 | 1 |
| 20. | I feel confident that I can learn about my clients' cultural background. | 6 | 5 | 4 | 3 | 2 | 1 |
| 21. | Cultural competence is included in my work place's mission statement, policies, and procedures. | 6 | 5 | 4 | 3 | 2 | 1 |
| 22. | I am effective in my nonverbal communication with clients whose culture is different from mine. | 6 | 5 | 4 | 3 | 2 | 1 |
| 23. | The way services are structured in my setting makes it difficult to identify the cultural values of my clients. | 6 | 5 | 4 | 3 | 2 | 1 |
| 24. | I feel that I have limited experience working with ethnic minority clients. | 6 | 5 | 4 | 3 | 2 | 1 |
| 25. | It is difficult to practice skills related to cultural competence. | 6 | 5 | 4 | 3 | 2 | 1 |
| 26 | I am sensitive to valuing and respecting differences between my cultural background and my clients' cultural heritage. | 6 | 5 | 4 | 3 | 2 | 1 |
| 27. | My workplace does not support using resources to promote cultural competence. | 6 | 5 | 4 | 3 | 2 | 1 |
| 28. | I have opportunities to learn culturally responsive behaviours from peers. | 6 | 5 | 4 | 3 | 2 | 1 |
| 29. | I do not feel that I have the skills to provide services to ethnic minority clients. | 6 | 5 | 4 | 3 | 2 | 1 |
| 30. | I examine my own biases related to race and culture that may influence my behaviour as a service provider. | 6 | 5 | 4 | 3 | 2 | 1 |
| 31. | I actively strive for an atmosphere that promotes risk-taking and self-exploration. | 6 | 5 | 4 | 3 | 2 | 1 |
| 32. | My workplace does not support my participation in cultural celebrations of my clients. | 6 | 5 | 4 | 3 | 2 | 1 |
| 33. | I would find it easy to work competently with ethnic minority clients. | 6 | 5 | 4 | 3 | 2 | 1 |
| 34. | I openly discuss with others issues I may have in developing multicultural awareness. | 6 | 5 | 4 | 3 | 2 | 1 |
| 35. | I learn about different ethnic cultures through educational methods and/or life experiences. | 6 | 5 | 4 | 3 | 2 | 1 |
| 36. | It is difficult for me to accept that religious beliefs may influence how ethnic minorities respond to illness and disability. | 6 | 5 | 4 | 3 | 2 | 1 |
